# Biomimicking Topographic Elastomeric Petals (E‐Petals) for Omnidirectional Stretchable and Printable Electronics

**DOI:** 10.1002/advs.201400021

**Published:** 2015-02-09

**Authors:** Ruisheng Guo, You Yu, Jifang Zeng, Xuqing Liu, Xuechang Zhou, Liyong Niu, Tingting Gao, Kan Li, Yong Yang, Feng Zhou, Zijian Zheng

**Affiliations:** ^1^ Nanotechnology CenterInstitute of Textiles and ClothingThe Hong Kong Polytechnic UniversityHong KongChina; ^2^ State Key Laboratory of Solid LubricationLanzhou Institute of Chemical PhysicsChinese Academy of SciencesLanzhou730000China; ^3^ University of the Chinese Academy of SciencesBeijing100049China; ^4^ Advanced Research Centre for Fashion and TextilesThe Hong Kong Polytechnic University Shenzhen Research InstituteShenzhen518000China; ^5^ Centre for Advanced Structural MaterialsDepartment of Mechanical and Biomedical EngineeringCity University of Hong KongTat Chee Avenue, Kowloon Tong KowloonHong KongChina

**Keywords:** biomimicking, electronic skins, polymer‐assisted metal deposition, printable electronics, stretchable electronics

## Abstract

**Elastomeric petals directly replicated from natural rose petal** are new versatile substrates for stretchable and printable electronics. Compared with conventional flat polydimethylsiloxane substrates, elastomeric petals have biomimicking topographic surfaces that can effectively inhibit the propagation of microcracks formed in the conducting layer, which is deposited on top, regardless of the type of conductive materials and the deposition methods.

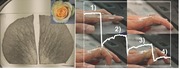

The booming of research in ultraflexible, stretchable, and wearable electronics in the past decade has witnessed the remarkable development of advanced materials,^1^, ^2^, ^3^ structures,^4^, ^5^, ^6^, ^7^, ^8^, ^9^ and devices^10^, ^11^, ^12^, ^13^, ^14^, ^15^, ^16^, ^17^, ^18^, ^19^ that can function under large tensile strains (1%). In particular, the realization of highly conductive and stretchable metal interconnects, contacts, and electrodes are recognized as one critical milestone for these thin film devices.^2^, ^13^, ^20^, ^21^, ^22^, ^23^, ^24^ Conventionally, metals are considered as rigid and nonstretchable materials due to their high Young's modulus, e.g., >100 GPa in the case of Cu.^25^ As a consequence, metal thin films deposited on plastic or flat elastic substrates crack seriously even when a small tensile strain was applied.^26^, ^27^ This challenge has been partially overcome by engineering planar metal thin films into wavy geometries, which make use of the reversible elastic deformation of wavy structures during stretch‐release activities to prevent cracking on metal. Two major wavy geometries, being buckle^28^, ^29^, ^30^, ^31^, ^32^, ^33^ and serpentine,^7^, ^21^, ^25^, ^34^, ^35^ have been reported the most to date. Metal buckles are fabricated by first depositing planar metal thin films or patterns on prestretched flat elastic substrates and subsequent release of the strain on elastomer to allow spontaneous buckling of metal.^29^ Metal serpentines are patterned directly on flat elastic substrates by lithographic techniques.^25^ On the basis of these wavy conductors, a wide range of stretchable electronic devices have been reported to date.

Nevertheless, these waves show several inherent drawbacks. 1) Sophisticated engineering design with multiple wavy structures is needed to achieve omnidirectional stretching ability. 2) In the fabrication of buckles, it is practically difficult to control the prestretching and strain release over large areas or on a specific site of a substrate. 3) The fabrication of serpentines involves many lithographic and etching steps, which is complicated and labor intensive. In addition, serpentines are mainly used as stretchable interconnects but are not suitable for electrode applications. These serpentine interconnects also inevitably decrease the device density. To date, the research community is still pursuing extensively for alternative strategies, which can avoid complicated fabrication and manipulation, while render conducting layers stretchable.^36^

To address the great challenge, we report herein a new biomimicking elastomeric petal (E‐petal), which can be used as versatile substrate for fabricating omnidirectional stretchable and printable metal conductors for thin film electronics, without the need for any prestretching or lithographic process. The E‐petals are made by highly scalable, one‐step soft lithographic replication using natural rose petals as molds. Instead of conventional flat elastomer substrates used in stretchable electronics, the upper surface of the E‐petal possesses continuous 3D microscale crater‐like topographies, of which the sharp ridges act as crack‐stopping edges. That is, when conducting materials such as metal thin films are deposited on top, the sharp ridges can effectively stop the propagation of microcracks in the conducting layer formed under large strains. As a consequence, the electrical resistance of the conducting layer shows remarkable stability in large‐strain deformation. As proof‐of‐concept, we demonstrate herein the fabrication of omnidirectional stretchable conductors using metal and conducting polymer deposited by vacuum deposition or solution casting methods. Finite element (FE) analysis is also performed to analyze the stretchable mechanism. More importantly, metal patterns can be readily printed onto the E‐petal by techniques such as inkjet printing. We demonstrate herein a fully printed strain sensor as electronic skins using interdigitated Cu electrodes, interconnects, and contacts on E‐petal. It should be noted that previous studies on biomimicking structures, such as lotus leaf and shark's skin,^37^, ^38^ have been largely focused on surface wetting/dewetting properties.^39^, ^40^ Only a few works reported the use of these relief structures to fabricate flexible electronic devices.^41^, ^42^ To the best of our knowledge, this is the first paper reporting substrates of biological topographic structures for stretchable electronics.

The fabrication of E‐petals followed one‐step soft lithography replication process as shown in **Figure** **1**a. In brief, fresh petals of yellow roses were taped onto a plastic petri dish and used as mold (Figure 1b,c). A mixture of polydimethylsiloxane (PDMS) prepolymer and its curing agent in a ratio of 10:1 was then poured onto the rose petals, followed by degassing in a vacuum desiccator. After curing at room temperature for 48 h, the E‐petals were obtained by peeling off the cured PDMS from the rose petal mold (Figure 1d). The thickness of the E‐petal was ≈1 mm.

**Figure 1:**
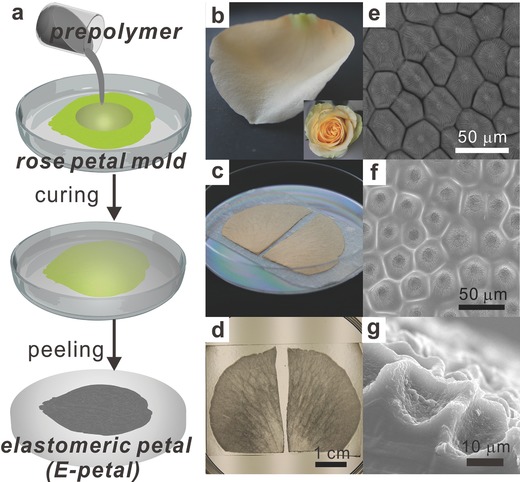
Preparation and characterization of E‐petals. a) Schematic illustration of the fabrication of E‐petals from natural rose petals. Digital images of b) a fresh yellow rose petal, c) rose petals taped on bottom of a petri dish, and d) as‐made E‐petals. SEM images of e) natural rose petal, f) topography, and g) cross‐section of E‐petals, respectively.

The surface of natural rose petals exhibited pentagonal and hexagonal micropapillaes that were 20–40 μm across and were separated by a web of trenches that were ≈2 μm wide (Figure 1e). On top of these micropapillaes, there were nanofolds that spread from the center to the edge. On the other hand, the surface of E‐petals showed an inverted structure to that of rose petal molds (Figure 1f,g). Microscale craters separated by a network of protruded ridges were observed on E‐petals, with lateral dimensions and shapes compatible with micropapillaes and microtrenches, respectively. The average height difference between the bottom of microcraters and the top of ridges was determined to be ≈15 μm. Nanofolds also appeared at the bottom of the microcraters, which indicates that the soft lithographic process can precisely replicate the micro and nanostructures from rose petals to E‐petals. Since the E‐petals were made of elastic PDMS, they could be readily stretched to 100% without mechanical failure and could return to their original dimensions without losing the micro‐ and nanostructures on the topographic surfaces.

It should be noted that each fresh rose petal mold could only be used for a few times for making E‐petals because shrinkage and distortion occurred as the water in the rose petal evaporated. Nevertheless, the first generation of E‐petals, i.e., E‐petals replicated from the natural rose petal molds, could be replicated by soft lithography again to produce second‐generation PDMS molds for future fabrication. We found that the surface topography of the second generation of the E‐petals using PDMS molds exhibited no obvious different from those directly made from fresh rose petal molds (Figure S1, Supporting Information) and the PDMS mold could be used for many times without losing its structural integrity and resolution.

E‐petals can be readily used for making solution‐processed stretchable metal conductors, which are critical and inevitable components in the future printable fabrication of highly flexible and stretchable electronics. Herein, we used a “polymer‐assisted metal deposition” (PAMD) method to form a uniform and conformal metal coating on E‐petals through a solution manner.^43^, ^44^, ^45^ In a typical experiment, a thin layer of poly[2‐(methacryloyloxy)ethyl‐trimethylammonium chloride] (PMETAC, ≈20 nm) was first grafted onto the topographic surface of E‐petals through surface‐initiated polymerization. Subsequently, ionic species [PtCl_4_]^2‐^ were immobilized onto the quaternary ammonium groups of PMETAC by immersing the PMETAC modified E‐petals into an aqueous solution of (NH_4_)_2_PtCl_4_. After brief rinsing with deionized (DI) water, the substrate was immersed into an electroless deposition (ELD) bath of Cu for 15 min at room temperature, in which a Cu thin film was formed. We denote this sample as ELD‐Cu/E‐petal in this paper (**Figure** **2**a).

**Figure 2:**
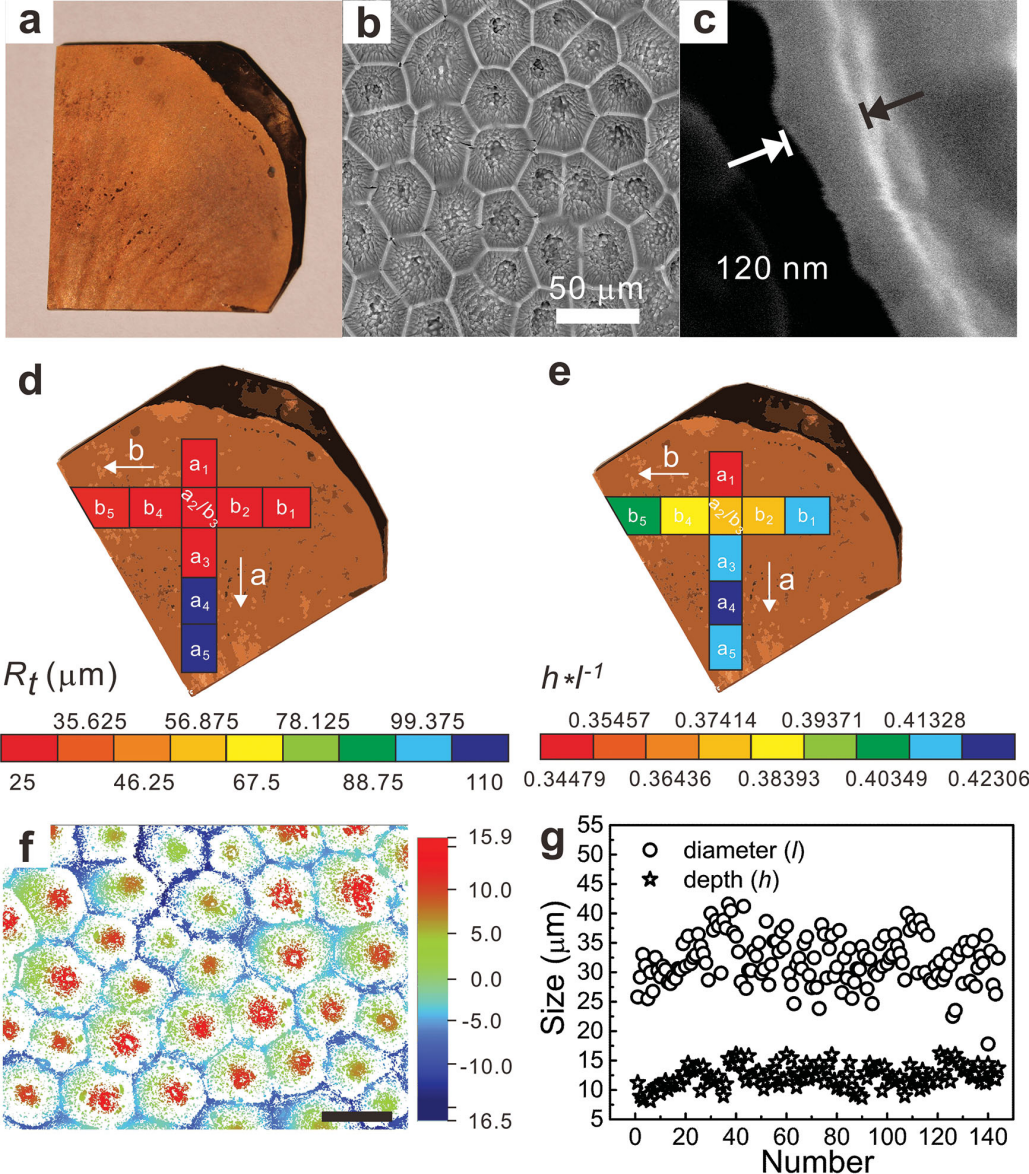
Characterization of ELD‐Cu/E‐petals. a) Digital image of ELD‐Cu/E‐petal. b) SEM image showing the topographic structure of ELD‐Cu/E‐petal. c) Cross‐sectional SEM image of ELD‐Cu/E‐petal, showing the thickness of Cu. d) *R_t_* and e) h*l ^‐1^ distribution along *a* and *b* directions. f) A typical surface profile mapping of ELD‐Cu/E‐petal. The scale bar is 30 μm. g) Distribution of diameter (*l*) and depth (*h*) of ELD‐Cu/E‐petal.

The metal coating of ELD‐Cu/E‐petal was uniform and continuous over the entire substrate. Topographic microcraters and nanofolds were clearly observed on the surface of ELD‐Cu/E‐petal, and the thickness of ELD‐Cu was ≈120 nm (Figure 2b,c). ELD‐Cu/E‐petal showed remarkable conductivity, being 2.0 × 10^7^ S m^−1^, which indicates that the Cu layer is highly compact. We studied the surface topographic structures of ELD‐Cu/E‐petal by surface profiler meter mapping at nine different spots as marked in Figure 2d,e. Arrows *a* and *b* indicated the directions along and perpendicular to the main vein of the petal, respectively. The maximum height of the profile (*R_t_*), diameter (*l*), and depth (*h*) of the microcraters were measured. Statistical analysis (Figure 2f,g) showed that they were 31.5 ± 4.0 μm across and 12.5 ± 1.5 μm deep. This surface morphology was very similar to that of the underlying E‐petal surface, which indicates that the PAMD method is ideal for making a conformal metal coating on the topographic surface.

Importantly, ELD‐Cu/E‐petal possessed stable electrical conductivity when it was bent or folded. For flexibility tests, ELD‐Cu/E‐petal was clamped on a home‐built uniaxial moving stage, and the two‐point electrical resistance was recorded when the samples were bent at different radius of curvatures (*r* = 7, 4, and 1 mm) for 5000 cycles each. It should be noted that when *r* = 1 mm, ELD‐Cu/E‐petal was fully folded. **Figure** **3**a shows the bending test results, in which *R*_0_ is the initial electrical resistance of sample before bending, and *R/R*_0_ is the normalized resistance during bending tests. When *r* = 7 mm, *R/R*_0_ increased slowly to 1.13 after 1000 bending cycles and remained stable until 5000 cycles. Similar phenomenon was also observed at other bending radius. Smaller bending radius resulted in slightly higher *R/R*_0_ after 5000 bending cycles, being 1.21 and 1.58 at *r* = 4 mm and *r* = 1 mm, respectively.

**Figure 3:**
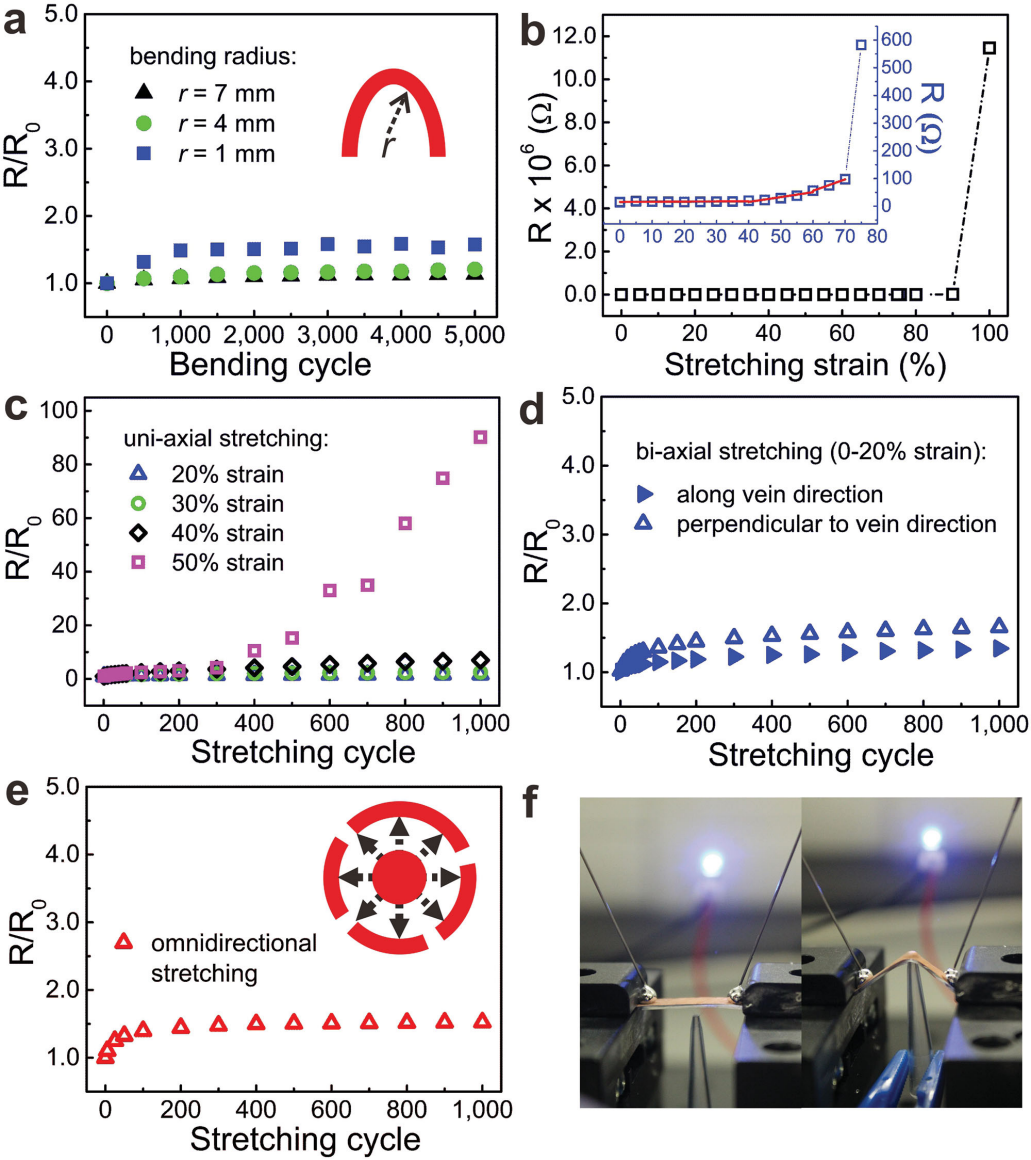
Flexibility and fatigue tests of ELD‐Cu/E‐petals under bending, uniaxial stretching, biaxial stretching, and omnidirectional stretching. a) Normalized resistances of ELD‐Cu/E‐petals during 5000 testing cycles at different radius of bending curvature (*r*). b) Resistance‐strain curve of ELD‐Cu/E‐petal stretched from 0% to 100% strain. Inset shows the partially magnified curve. c) Normalized resistances of ELD‐Cu/E‐petals during 1000 cycles of uniaxial tensile tests with different maximum strains (20%, 30%, 40%, and 50%). d) Normalized resistances of ELD‐Cu/E‐petals during 1000 cycles of biaxial tensile tests. e) Normalized resistance of ELD‐Cu/E‐petals during 1000 cycles of omnidirectional tensile tests. f) Digital images of a LED circuit using ELD‐Cu/E‐petal interconnect with (right) and without (left) punching. The light intensity of the LED indicated that the current was stable even when ELD‐Cu/E‐petal was punched significantly.

More importantly, ELD‐Cu/E‐petal also acquired remarkable properties as high‐performance stretchable conductors. We first measured the electrical resistance of ELD‐Cu/E‐petal during one cycle of 0–100% strain (along vein direction, *a*) uniaxially in order to probe its affordable strain range. It was observed that its electrical resistance maintained stable until 40% strain and then rose slowly between 40% and 70% strain, followed by a steep increase until 100% strain (Figure 3b). The electrical conductance was completely lost when the strain was larger than 90%. This phenomenon implies that 40% strain is a critical point for ELD‐Cu/E‐petal. We then carried out detail stretchability study by conducting a series of 1000‐cycle stretch‐release deformations on the ELD‐Cu/E‐petal with different maximum strains. When stretching tests were carried out below 40% strain, i.e., tests at 0–20%, 0–30%, and 0–40% strain cycles, ELD‐Cu/E‐petal showed satisfactory properties as stretchable conductors (Figure 3c). Its resistance increased slightly at the first 300 cycles and then maintained relatively stable afterwards. The final *R/R*_0_ values after 1000‐cycle tests were 1.6, 2.4, and 6.9, respectively (Figure 3c). However, for 0–50% strain cycles, *R*/*R*_0_ increased continuously to 90 after 1000 cycles. It was very likely that further increase in electrical resistance would occurred if more stretching cycles were performed. Uniaxial stretching tests perpendicular to the vein direction (arrow *b*) also showed similar results. For example, after 1000 cycles of 0–20% strain tests, *R*/*R*_0_ increased to 1.4, which was similar to that of the vein direction (Figure 3d). This result indicates that ELD‐Cu/E‐petal can be used as biaxial stretchable conductors. In contrast, when the 120 nm thick Cu thin film was deposited on a flat PDMS substrate by the same PAMD process (sample denoted as ELD‐Cu/flat‐PDMS), its electrical resistance went up steeply by 1 × 10^7^ folds even when a very small (1–2%) tensile strain was applied (Figure S2, Supporting Information).

Apart from uniaxial and biaxial stretchability, we also tested the omnidirectional stretchability of ELD‐Cu/E‐petal conductors. The sample was clamped with a pair of stainless steel rings and a stainless steel ball was punched into the center of ELD‐Cu/E‐petal to induce omnidirectional stretching (Figure 3e). The average maximum strain on the sample was calculated to be 13% in accordance to a literature method.^46^ Further increase in punching force would break the substrate at the clamped positions. Similar to the trends observed in uniaxial and biaxial tests, *R*/*R*_0_ of the omnidirectional stretching test increased to 1.5 after the first 300 cycles and then maintained stable until 1000 cycles. Such a high‐performance omnidirectional stretchability without the need of any lithographic or prestretch treatment is the most important characteristic of ELD‐Cu/E‐petal. Note that conventional wavy buckles or serpentines fabricated on flat elastomeric substrates required multistep fabrications. In addition, complicated pattern design was needed to achieve omnidirectional stretchability.

These omnidirectional stretchable ELD‐Cu/E‐petal conductors are highly suitable for wearable and stretchable electronic applications, in which nonregular 3D deformations occur frequently. As a proof‐of‐concept demonstration, we integrated a light‐emitting diode (LED) into an electronic circuit with ELD‐Cu/E‐petal interconnects. As shown in Figure 3f, a DC voltage of 3 V was applied while the ELD‐Cu/E‐petal interconnect was punched by a sharp pipette tip. It could be seen that the LED intensity did not have obvious change even though the ELD‐Cu/E‐petal deformed largely under punching.

Note that the easy fabrication of stretchable conductors on E‐petal by solution‐processed methods is ideal for printable electronics applications. As proof‐of‐concept, we developed a strain sensor using printed stretchable metal electrodes on E‐petal for use as electronic skins. As shown in **Figure** **4**a, six pairs of interdigitated electrodes with interconnects and contacts made of a bilayer of Cu and Ag (Ag on top of Cu) were first printed on E‐petal by inkjet printing. Briefly, E‐petal was modified with PMETAC as discussed above. Then [PdCl_4_]^2‐^ containing ink was inkjet printed onto E‐petal according to the pattern design, followed by consecutive ELD of Cu and Ag to form the bilayered Ag/Cu electrodes, interconnects and contacts. The width and the gap of the electrodes were 650 and 350 μm, respectively. Subsequently, graphene oxide (GO) was spin‐coated onto the Ag/Cu electrodes and was reduced to form a reduced GO (rGO) thin layer. The initial resistances of the device measured at the two contact pads were 1.2 KΩ at 0% strain and 1.9 KΩ at 20% strain. When the device was stretched, the resistance at 0% remained unchanged, while that at 20% strain increased to 2.2 folds after the first 300 tensile cycles and then stabilized thereafter (Figure 4b). At this point, the current (under a constant bias) was a first‐order function of the tensile strain. Therefore, we could use this device as a strain sensor by monitoring the current change of the device (Figure 4c). For example, we attached the device onto a joint of the index finger and measured the current change at four different bending gestures of the finger (Figure 4d). The current dropped as finger bent, which was due to the large tensile strain applied on the sensor. Four different steps were observed during the test, presenting the four bending gestures of the finger (Figure 4e). Such a testing could be repeated many times with very good reproducibility.

**Figure 4:**
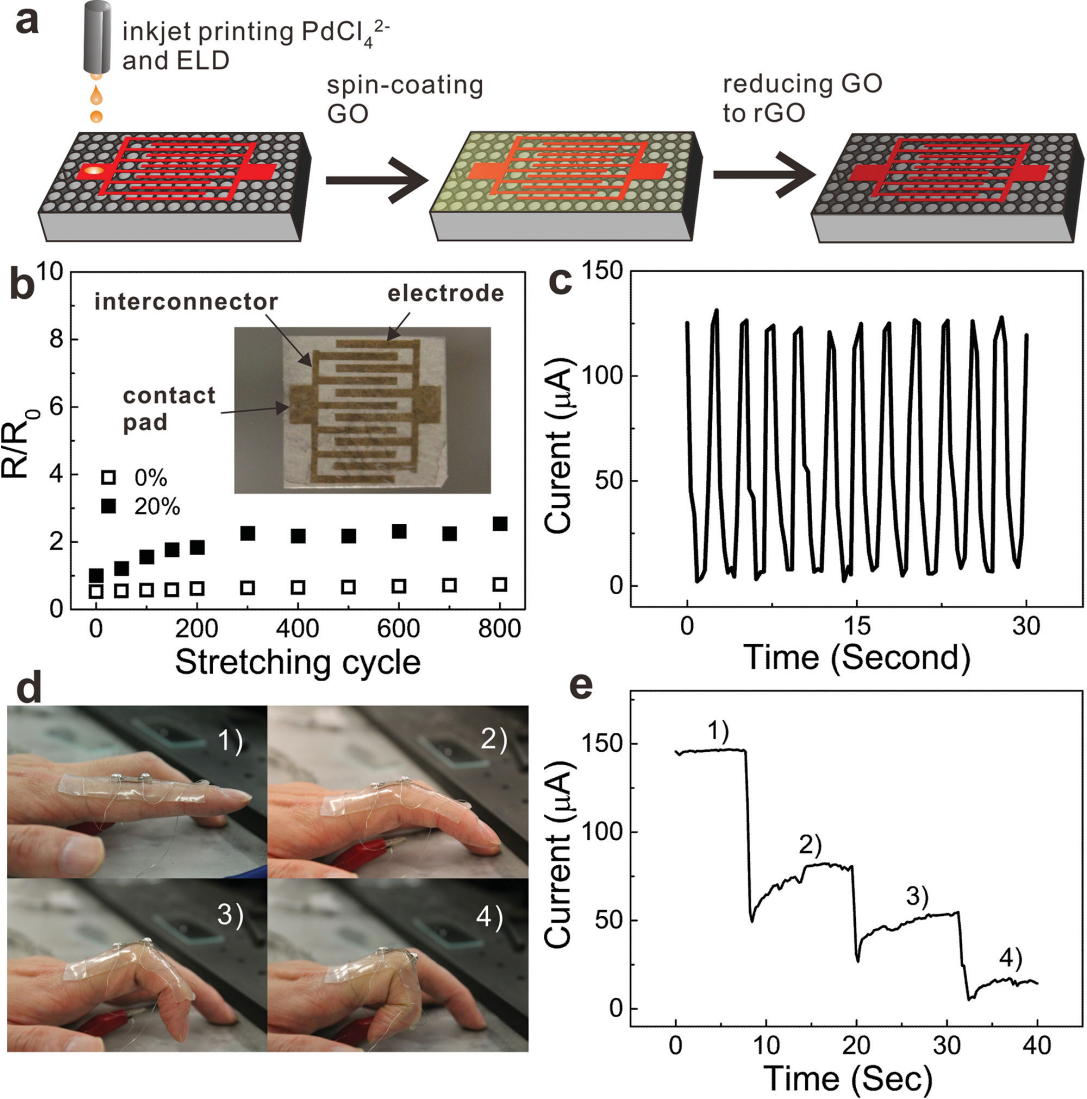
Printed strain sensors on E‐petal as electronic skins. a) Schematic illustration of the fabrication process of printed strain sensors on E‐petal. b) Normalized resistances of the strain sensor during 800 cycles of tensile tests. Inset: digital image of the device. c) Magnified current change of the device during the tensile tests. d) Demonstration of the printed strain senor as electronic skin. The device was attached on a finger, which was bent at four different gestures. e) Typical current change of the strain sensor at the four different states shown in (d).

To understand the much superior performance of ELD‐Cu/E‐petal to ELD‐Cu/flat‐PDMS, we observed the surface morphologies of both samples after the tensile tests. Owing to the mechanical mismatch between the hard Cu thin film and soft elastomeric substrate, cracking of the Cu layer may occur even when the applied tensile strain is small. Such surface cracking usually propagates rapidly once initiated, leading to the breakage of conductive pathway of electrical currents. This was what happened to ELD‐Cu/flat‐PDMS: a large number of long and large cracks on the Cu layer were easily observed by naked eyes even just after one cycle of 2% strain deformation. On the contrary, only short and small cracks were found under electron microscopy on ELD‐Cu/E‐petal that was stretched for 1000 cycles with a maximum strain of 40% (**Figure** **5**a–c). These small cracks mostly existed at the bottom and sidewall of the microcraters and were separated by the sharp ridges between the microcraters. The density of these cracks was low, which did not reach the percolation limit to effectively block the electron conduction in the Cu film. As a result, ELD‐Cu/E‐petal maintained a high electrical conductivity even at a high tensile strain.

**Figure 5:**
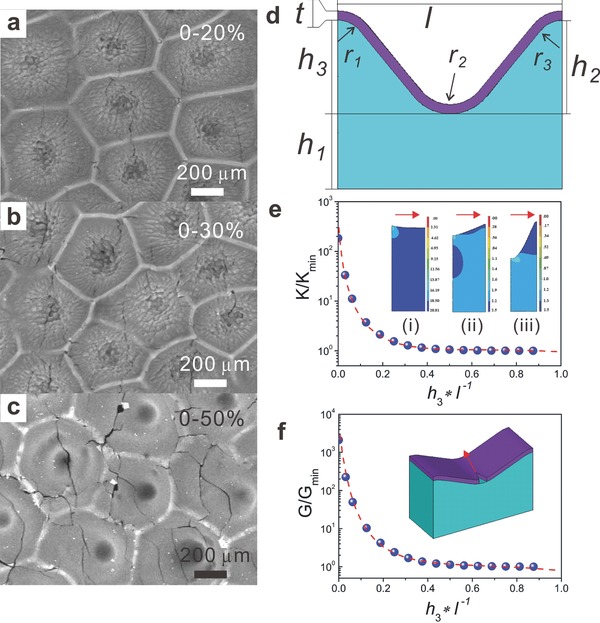
Characterization of stretched ELD‐Cu/E‐petals and FE simulation study. a–c) SEM images of ELD‐Cu/E‐petals after 1000 cycles of tensile tests at different maximum strains. d) Schematic cross‐section of the simulation cell extracted from the surface profile image (not in scale). e) The normalized stress intensity factor for the film crack to penetrate into the substrate at different *h*_3_*/^‐1^(strain = 30%). The insets are the Von Mises total strain contours for three *h*_3_*/^‐1^ ratios: i) 0.03, ii) 0.25, and iii) 0.88. The red arrows show the strain directions. *K*_min_ = 0.53 MPa m^0.5^ for *h*_3_*/^‐1^ = 0.88. K is stress intensity factor, which is a measure of how much energy being offered for crack growth. (f) The normalized energy release rates for surface cracks at different *h*_3_*/^‐1^ (strain = 30%). For *h*_3_*/^‐1^ = 0.88, *G*_min_ =120 J m^‐2^. The inset is the sketch of a surface crack in the copper film on a PDMS substrate and the red arrow shows the propagation direction of the surface crack.

The remarkable inhabitation of crack propagation is attributed to the biomimicking topography of E‐petals, as revealed by our FE simulation study. According to literature, our simulations were focused on the two worst‐case scenarios that contributed to the tension‐induced cracking in thin films: surface cracking and substrate damage.^47^ To simplify our analysis, we treated the 3D cracking problem as a series of 2D problems, each of which corresponded to cracking in one particular cross section of the well‐like cell structure (Figure 5d). Taking the surface topography of ELD‐Cu/E‐petal into consideration, we fixed *r*_1_ = *r*_3_ =* r*_0_, *r*_2_ = 1.8*r*_0_, *h*_2_ = *h*_3_ throughout the simulations and varied *h*_3_ */l* to investigate different surface morphologies (Figure 5e). Both the film and the substrate were taken to be linearly elastic with *E_f_* = 100 GPa, *n_f_* = 0.3, *E_s_* = 1 MPa, and *n_s_* = 0.49, where the symbols *E* and *n* stand, respectively, for elastic modulus and Poisson's ratio and the subscript *f* and *s* stand, respectively, for Cu thin film and PDMS substrate. For surface cracking, the thermodynamic driving force for fracture was the elastic energy released as a surface crack grew within the film. Evidently, as *h*_3_ */l* increased from nearly zero (corresponding to a flat surface) to one (corresponding to an E‐petal surface), *G* plunged by three orders of magnitude (Figure 5f). For substrate damage, the elastic energy release rate decreased significantly with the increasing *h*_3_/*l*, which indicates that film cracks are difficult to penetrate into the polymer substrate when they propagate to the ridge regions of the microcraters.

According to the above FE simulation results, it can be seen that the film cracks, even though easily initiated from the regions of large stress concentration, cannot grow with ease due to the surface microstructure effect. This behavior is fully consistent with our experimental observations. At the bottom of microcraters of ELD‐Cu/E‐petal, cracks on Cu were easily formed during strain deformations due to the relatively flat geometry. However, the growth of surface cracks became extremely difficult once they extended from the bottom of the microcraters of E‐petal to the boundary ridges. Therefore, the microcracks on ELD‐Cu/E‐petal remained small and low‐density during the tensile tests, as we observed from the morphology study. Because the microcraters were arranged continuously all over the surface, ELD‐Cu/E‐petal possessed remarkable omnidirectional stretchability. However, when the tensile strain was too large (>50%), the E‐petal topography was not able to fully stop the crack propagation. As a result, the electrical resistance kept rising throughout the entire test.

The FE simulation above indicates that E‐petal should be a universal substrate for making many kinds of stretchable conductors, provided that the conductive materials can be deposited conformably as a thin film on E‐petal with good adhesion. As proof‐of‐concept, we demonstrated two more stretchable conductors on E‐petals using Cu made by thermal evaporation (EVP‐Cu/E‐petal) and poly(3,4‐ethylenedioxythiophene):poly(styrene sulfonate) made by spin‐coating(PEDOT:PSS/E‐petal). For comparison, we also deposited the same materials on flat PDMS under the same conditions and denoted them as EVP‐Cu/flat‐PDMS and PEDOT:PSS/flat‐PDMS.

Before thin film deposition, an oxygen plasma treatment (3 min) on the substrates (both E‐petal and flat PDMS) was performed to render the surface hydrophilic. For EVP‐Cu/E‐petal, a thin Ti adhesion layer was evaporated prior to the evaporation of Cu, and the total thickness of the Ti/Cu bilayer was maintained to be 120 nm, which was as thick as the Cu thin film of ELD‐Cu/E‐petal. The surface topography of EVP‐Cu/E‐petal was very similar to that of ELD‐Cu/E‐petal, showing a good conformal coating of Cu on the surface of E‐petal. **Figure** **6**a shows the tensile test (0–20%) results of EVP‐Cu/E‐petal with different Ti layer thicknesses. Without the Ti layer, serious cracking and delamination of the evaporated Cu thin film occurred when EVP‐Cu/E‐petal was stretched, which was due to the lack of adhesion between Cu and E‐petal. When a thin Ti layer was applied, EVP‐Cu/E‐petal exhibited remarkable stretchability, which was similar to that of ELD‐Cu/E‐petal. After 1000 tensile tests, the electrical resistance increased by only threefolds, when the thickness of Ti layer was 5–15 nm thick. However, when the thickness of Ti was further increased to 25 nm, the increase in electrical resistance became more significant, indicating that a thin Ti was more suitable. For the case of PEDOT:PSS/E‐petal, it was found that the normalized electrical resistance value maintained stably at 1 during the 1000‐cycletest (Figure 6b). This was because the Young's modulus of PEDOT:PSS (a few GPa) was much lower than Cu (100 GPa) and no crack formation was observed on PEDOT:PSS/E‐petal. On the contrary, EVP‐Cu/flat‐PDMS was not conductive at all even just stretched to 1% strain due to serious cracking of Cu. PEDOT:PSS/flat‐PDMS was not stretchable after one stretch to 18% strain, its electrical resistance went up by seven orders of magnitudes (Figure 6c).

**Figure 6:**
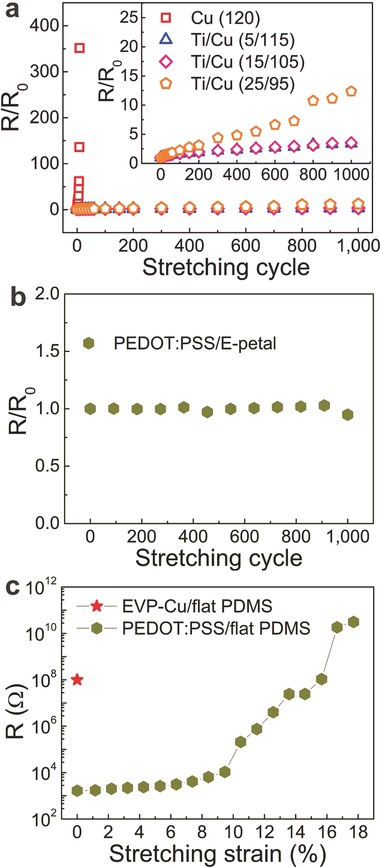
E‐petal as a universal substrate for various stretchable conductors. a) Normalized resistances of EVP‐Cu/E‐petals with different thickness of the Ti adhesion layer (0, 5, 15, and 25 nm) during 1000 cycles of uniaxial tensile tests (0–20%). b) Normalized resistance of PEDOT:PSS/E‐petal during 1000 cycles of uniaxial tensile tests (0–20%). c) Resistance changes of EVP‐Cu/flat‐PDMS (10 nm Ti/110 nm Cu) and PEDOT:PSS/flat‐PDMS during one cycle of tensile test (0–18%).

In conclusion, we reported the development of E‐petal, a new biomimicking elastomeric substrate, which is suitable for making a wide variety of stretchable conductors and devices. The most enabling characteristic of E‐petal, as revealed by our experimental and simulation analysis, is its 3D topographic surface, which can effectively stop the crack propagation in the conducting materials (deposited on top of the E‐petal) under large strains. It should be noted that there are a few attempts to use rough PDMS substrates to fabricate stretchable conductors without prestretching or serpentine patterning in the literature. Lambricht et al. fabricated irregular rough Au/PDMS with rough glass side molds, but its resistance change was more than 100 folds.^36^ Apaydin et al. obtained Cu thin film with pyramid tips on PDMS/ceramic composites using microtextured nylon molds but microcracks on the Cu thin film were found just after evaporation of Cu.^48^ Park et al. obtained periodic 1D and 2D wavy metal structures on PDMS stamp replicated from pattern‐etched Si molds, but they could only be stretched uniaxially or biaxially when the tensile strain was less than 20%.^49^ Compared with these literature reports, the ELD‐Cu/E‐petal shows obvious advantages in the stretchability range, the omnidirectional stretching capability, and the ease in fabrication.

E‐petal processes several remarkable advantages far surpassing other conventional substrates for stretchable electronics as follows. 1) E‐petal can be directly used for making omnidirectional stretchable conductors without the need for any prestretching of substrate or complicated serpentine design and patterning, which is required when using conventional flat PDMS substrates. 2) As a consequence, E‐petal is suitable for roll‐to‐roll printable electronics because the patterning and materials deposition process does not require special handling, alignment or design. We have demonstrated a printed strain sensor for electronic skin applications. 3) E‐petal is versatile for many kinds of conductive materials such as metal and conducting polymer, regardless of their deposition methods. The only requirement is to deposit the conducting materials conformably on the E‐petal surfaces. For example, we have demonstrated Cu thin films deposited by either solution‐processed or vapor deposition methods are highly stretchable. 4) The fabrication of E‐petals itself is very simple. It only requires one‐step soft‐lithographic replication from natural rose petals. One can also use second‐generation PDMS molds for making the E‐petals without losing the topographic structure and resolutions. At this moment, the size of E‐petals is limited to the size of rose petals (≈2 × 2 inches), which is still small for large‐area applications. In the future, the size of E‐petals may be scaled up by using large man‐made molds (fabricated by lithographic methods such as photolithography) with topographies similar to natural rose petals. We believe that E‐petals can replace flat PDMS substrates in many applications for making stretchable conductors and devices in the future.

## Experimental Section

*Preparation of E‐Petals*: PDMS prepolymer and the curing agent were mixed in a ratio of 10:1 and the bubbles were removed by degassing in a vacuum desiccator. The mixture was poured onto the surface of rose petals, which were taped on a plastic petri dish. After being cured at room temperature for 48 h, textured PDMS was peeled off from rose petal. Then PDMS was treated by ultrasonication in NaOH aqueous solution (13 wt%), acetone, ethanol, and DI water in sequence. Finally, the biomimicking E‐petal was formed after curing at 70 °C for 2 h in an oven.

*ELD of Cu on PDMS*: E‐petals or flat stamps were exposed to an oxygen plasma for 3 min at 1300 mbar, followed by spin‐coating an [3‐(methacryloyloxy)propyl]trimethoxysilane ethanol solution (1:1, v/v) at 3000 rpm for 30 s. The substrates were incubated in an ammonia (90% humidity) atmosphere to accelerate the hydrolysis of saline for ≈6 h at room temperature. The modification of PMETAC with vinyl‐group were conducted by immersing the substrates into the mixture of METAC solution (80 wt% aqueous solution, Sigma‐Aldrich), DI water and potassium peroxodisulfate (Sigma‐Aldrich) (5 g:15 g:50 mg), and then polymerizing at 80 °C for 30 min. After being washed with DI water and dried in air, the PMETAC‐modified E‐petal and flat PDMS substrates were obtained. Hereafter, they were immersing into 5 × 10^−3^ m L^−1^ (NH_4_)_2_PdCl_4_ (Sigma‐Aldrich) aqueous solution for 15 min for loading PdCl_4_^2‐^ into PMETAC polymer. The ELD of Cu was performed in a plating bath consisting of a 1:1 (vol%) mixture of freshly prepared solution A and B. Solution A contained 12 g L^−1^ NaOH (Uni‐Chem), 13 g L^−1^ CuSO_4_·5H_2_O (Uni‐Chem), and 29 g L^−1^ KOCOCH(OH)CH(OH)COONa·4H_2_O (Uni‐Chem) in DI water. Solution B is a 9.5 mL L^−1^ HCHO (Uni‐Chem) aqueous solution. After ELD, the Cu/E‐petal was obtained by rinsing and drying.

*Thermal Evaporation of Cu on PDMS*: Before evaporating Cu thin films, E‐petal and flat PDMS surfaces were treated by oxygen plasma and then Ti were evaporated at a rate of 0.08 nm s^−1^. Cu thin films were evaporated at a rate of 0.2 nm s^−1^. Both layer deposition of Ti and Cu were conducted in a high vacuum environment with pressure of 5 × 10^‐4^ Pa.

*Spin‐Coating of PEDOT:PSS*: As the case of PEDOT:PSS/E‐petal, we used Bao's recipe to spin‐coat PEDOT:PSS on E‐petal.^50^ After activating the PDMS surfaces by oxygen plasma, we spin‐coated PEDOT:PSS (H.C. Starck, Clevios PH1000, mixed with 5 wt% dimethylsulfoxide, and 0.5 wt% Zonyl FS‐300 fluorosurfactant, obtained from Fluka) at 1000 rpm for 60 s, followed by annealing at 150 °C for 15 min.

*FE Simulation*: According to the literature, tension‐induced cracking in thin films could take place in a variety of forms, including surface cracking, channeling, substrate damage, spalling, and debonding.^47^ However, given the same external conditions, such as film thickness, applied stress and elastic modulus, surface cracking and substrate damage always give rise to the highest crack driving force.^47^ Therefore, our simulations were focused on the two worst‐case scenarios: surface cracking and substrate damage. FE simulations were conducted using the commercial package ANSYS. Because of symmetry, only one half geometry of Figure 1a was built into the FE model. Furthermore, both the film and substrate are taken to be linearly elastic with *E_f_* = 100 GPa, *n_f_* = 0.3, *E_s_* = 1 MPa, and *n_s_* = 0.49, where the symbols *E* and *v* stand, respectively, for elastic modulus and Poisson's ratio and the subscript *f* and *s* stand, respectively, for film and substrate. For the case of surface cracking the thermodynamic driving force for fracture is the elastic energy released as a surface crack grows within the film. Therefore the energy release rate, *G*, can be calculated to be 
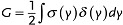
 according to literature,^51^ where *y* is the coordinate normal to the film, *σ*(*y*) is the elastic stress acting on the “crack” faces before they separate from each other while *δ* (*y*) is the opening displacement after cracking. To compute *G* entails two elastic FE simulations: one is to obtain the stress *σ*(*y*) without cracking and the other is to obtain the opening displacement *δ* (*y*) after cracking.

*Fabrication of Printed Strain Sensors*: (NH_4_)_2_PdCl_4_ (14 mg) and 10 mg poly(ethylene glycol) (PEG, *M*_w_ 4000, Uni‐Chem) were dissolved in 10 g DI water as inkjet inks. Samples were placed into the chamber of an inkjet printer (Jetlab 4, Microfab Technologies, Inc.). Inks were printed following patterns of electrodes, interconnects, and contact pads and were remained on the substrate for several minutes for loading PdCl_4_^2–^ into PMETAC polymer. Bilayered Ag/Cu was deposited by ELD of Cu and Ag, consecutively. The Ag plating bath consisted of 1 g L^−1^ [Ag(NH_3_)_2_]NO_3_ (Uni‐Chem) and 5 g L^−1^ KOCOCH(OH)CH(OH)COONa·4H_2_O. Finally, GO solution, which was prepared by modified hummers' method, was spin‐coated on the electrodes/E‐petal surface at 300 rpm for 20 min. Then GO was reduced in hydrazine vapor atmosphere overnight in fume hood followed by subsequent annealing at 90 °C.

*Bending and Stretching Tests*: Bending tests, uniaxial and biaxial tensile tests were conducted by home‐built and motorized uniaxial stretcher. Omnidirectional tensile test was conducted using ball‐punch Instron5565A. The bulk resistances were measured by two‐point probe method with a Keithley 2400 Sourcemeter and collected by computer‐controlled software.

*Other Characterization*: Morphologies of samples were investigated by scanning electron microscope (SEM, Hitachi TM3000; JEOL Model JSM‐6490). Surface profiles were measured by optical surface profiler (Wyko NT9300, Veeco).

## Supporting information

As a service to our authors and readers, this journal provides supporting information supplied by the authors. Such materials are peer reviewed and may be re‐organized for online delivery, but are not copy‐edited or typeset. Technical support issues arising from supporting information (other than missing files) should be addressed to the authors.

Supplementary
