# Supplementary material for: Biomimicking Topographic Elastomeric Petals (E‐Petals) for Omnidirectional Stretchable and Printable Electronics
Source: Adv Sci (Weinh). 2015 Feb 9;2(3):1400021. doi: 10.1002/advs.201400021 (PMC5115289; doi:10.1002/advs.201400021)
Supplement: Supplementary file 1 — Supplementary [file ADVS-2-0b-s001.pdf]

Copyright WILEY-VCH Verlag GmbH & Co. KGaA, 69469 Weinheim, Germany, 2015.

Supporting Information

for *Adv. Sci.,* DOI: 10.1002/advs.201400021

Biomimicking Topographic Elastomeric Petals (E-Petals) for Omnidirectional Stretchable and Printable Electronics

Ruisheng Guo, You Yu, Jifang Zeng, Xuqing Liu, Xuechang Zhou, Liyong Niu, Tingting Gao, Kan Li, Yong Yang, Feng Zhou, and Zijian Zheng**

Copyright WILEY-VCH Verlag GmbH & Co. KGaA, 69469 Weinheim, Germany, 2013.

Supporting Information

Bio-mimicking elastomeric petals as versatile substrates for omnidirectional stretchable and printable electronics

Ruisheng Guo[1,2,5], You Yu[1,3], JifangZeng[4], Xuqing Liu[1,3], Xuechang Zhou[1,3], Liyong Niu[1,3], Tingting Gao[1,2,5], Kan Li[1], Yong Yang[4], Feng Zhou[2,], Zijian Zheng[1,3,]**

[*] Prof. Zijian Zheng, Dr. Ruisheng Guo, Dr. You Yu, Dr. Xuechang Zhou, Dr. Tingting Gao, Xuqing Liu, Liyong Niu, Kan Li
1. Nanotechnology Center, Institute of Textiles and Clothing, The Hong Kong Polytechnic University, Hong Kong, China
Fax: (+) 852-27731432
E-mail: tczzheng@polyu.edu.hk

Prof. Feng Zhou, Dr. Ruisheng Guo, Dr. Tingting Gao
2. State Key Laboratory of Solid Lubrication, Lanzhou Institute of Chemical Physics, Chinese Academy of Sciences, Lanzhou 730000, China
Email: zhouf@licp.cas.cn

Prof. Zijian Zheng, Dr. You Yu, Dr. Xuechang Zhou, Xuqing Liu, Liyong Niu
3. Advanced Research Centre for Fashion and Textiles, The Hong Kong Polytechnic University Shenzhen Research Institute, Shenzhen 518000, China

Prof. Yong Yang, Dr. Jifang Zeng
4. Centre for Advanced Structural Materials, Department of Mechanical and Biomedical Engineering, City University of Hong Kong, Tat Chee Avenue, Kowloon Tong, Kowloon, Hong Kong, China

Dr. Ruisheng Guo, Dr. Tingting Gao
5. University of the Chinese Academy of Sciences, Beijing, 100049, China

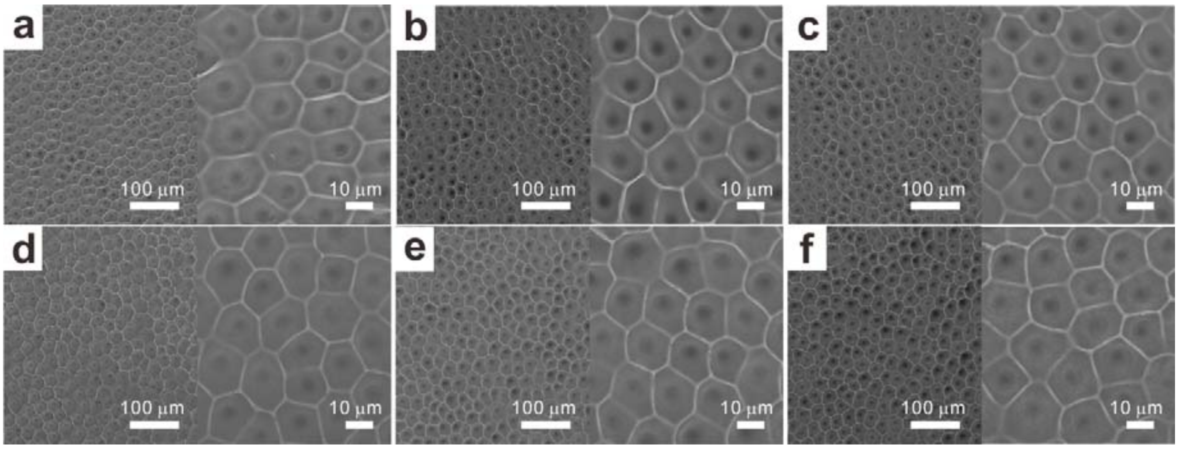

Figure S1. SEM imagesat different magnification scales of E-petal replicated from (a) nature rose petal mold and (b-f) the second-generation PDMS mold. (b): 2nd replication;(c): 4th replication; (d) 6th replication;(e) 8th replication; (f) 10th replication.

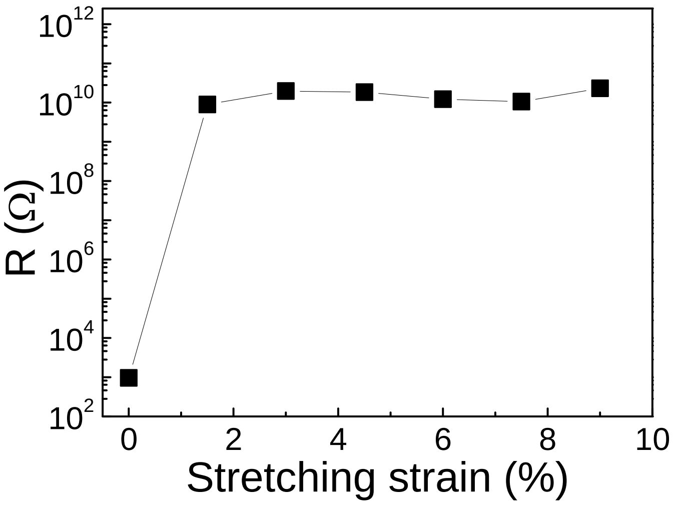

Figure S2. Resistance changes of ELD-Cu/flat-PDMS with increasing strains.
